# Flexibility and resistance exercises versus usual care for improving pain and function after distal radius fracture in adults aged 50 years or over: protocol for the WISE randomised multicentre feasibility trial

**DOI:** 10.1186/s40814-022-01011-5

**Published:** 2022-03-07

**Authors:** David J. Keene, Cynthia Srikesavan, Juul Achten, Elizabeth Tutton, Susan J. Dutton, Ioana R. Marian, Richard Grant, Jenny Gould, Kate Herbert, Amrita Athwal, Duncan Appelbe, Sarah E. Lamb, Matthew L. Costa

**Affiliations:** 1 Nuffield Department of Orthopaedics, Rheumatology and Musculoskeletal Sciences, University of Oxford, Oxford, UK; 2 Patient and Public representative, Oxford, UK; 3 College of Medicine and Health, University of Exeter, Exeter, UK

**Keywords:** Distal radius fracture, Pain, Function, Exercise, Adherence, Quality of life

## Abstract

**Background:**

Distal radius fractures represent about 1 in 5 of all fractures treated in UK hospitals. Most distal radius fractures occur in women aged 50 years or over after a fall. Distal radius fractures are managed using splints or casting, some are also treated with surgical fixation. Patients often experience long-term muscle weakness of the hand and arm that may impact their ability to do daily activities such as personal hygiene, routine household chores and food preparation. We propose a structured and tailored flexibility and resistance exercise programme for the hand and arm supplemented with behaviour change strategies to help perform daily exercise. The main aim of our study is to assess the feasibility of conducting a definitive randomised controlled trial.

**Methods:**

This study is a multicentre, parallel-group individually randomised feasibility trial. We will recruit a minimum of 72 adults aged 50 years or over with distal radius fracture treated surgically or non-surgically from at least three UK National Health Service (NHS) hospitals. They will be randomised 1:1:1 to receive usual care, usual care and independent exercise with a single therapy session or usual care and supervised exercise with three therapy sessions over 12 weeks. Our primary feasibility objectives are (1) patient engagement assessed by recruitment, (2) acceptability of the interventions assessed by adherence and patient and clinician experience and (3) retention of participants in the trial. Outcome measures will be assessed at baseline, 3 months and at 6 months after randomisation. A qualitative sub-study will explore the experiences of the trial participants and therapists delivering the exercises.

**Discussion:**

A definitive trial will be considered feasible without major modifications if our progression criteria are met. If successful, the findings will inform the design of a future definitive RCT to evaluate the clinical and cost-effectiveness of the WISE exercise programme.

**Trial registration:**

ISRCTN12290145.

## Background

There are 100,000 distal radius fractures a year in the UK, representing 18% of all fractures seen in hospitals [1]. Most distal radius fractures occur in women aged 50 years or over [2] after reaching out a hand as a protective response to a fall [3]. Initial fracture management is by non-surgical management using splints or casting or, for more complicated fractures, by surgical fixation followed by splints or casting.

Immediately after initial treatment of the fracture, when the cast or splint is removed, the vast majority of patients experience pain and stiffness in their wrist and have muscle weakness of the upper limb, making self-care and activities of daily living difficult [4]. Exercises are often prescribed to improve recovery after distal radius fracture [5]. A recent systematic review, focussing on prescribed exercise after distal radius fracture, found that across nine trials there was insufficient evidence to (i) support referral for therapy, (ii) support patients with exercise progression and (iii) support the use of specific types of exercise, such as resistance training [6].

Current guidance for rehabilitation, in the absence of evidence, reflects common practice in the UK, which is advice from a surgeon or a physiotherapist on self-management [7]. The advice usually includes basic upper limb mobilising exercises to help recover joint range of motion, and provision of a patient information leaflet. Referral to physiotherapy or specialist hand therapy for supervised rehabilitation and specialist splinting is variable and often reserved for the minority of patients who experience serious complications [7].

Current exercise advice focuses on joint flexibility and on graded increase in use of the hand in day-to-day activities. We hypothesise that introducing structured, self-managed resistance exercise training has the potential to improve functional recovery by optimising recovery of muscle strength of the hand and upper limb. In the WISE feasibility trial (Wrist Injury Strengthening Exercise), we propose two different modes of delivering structured flexibility and resistance exercises (independent and supervised) with behaviour change strategies to support self-management. The primary objectives of this study are to determine the feasibility of conducting a definitive, multicentre RCT of structured flexibility and resistance exercise compared to usual care, in improving pain and function after distal radius fractures in adults aged 50 years and over.

## Methods/design

### Trial design

**Fig. 1:**
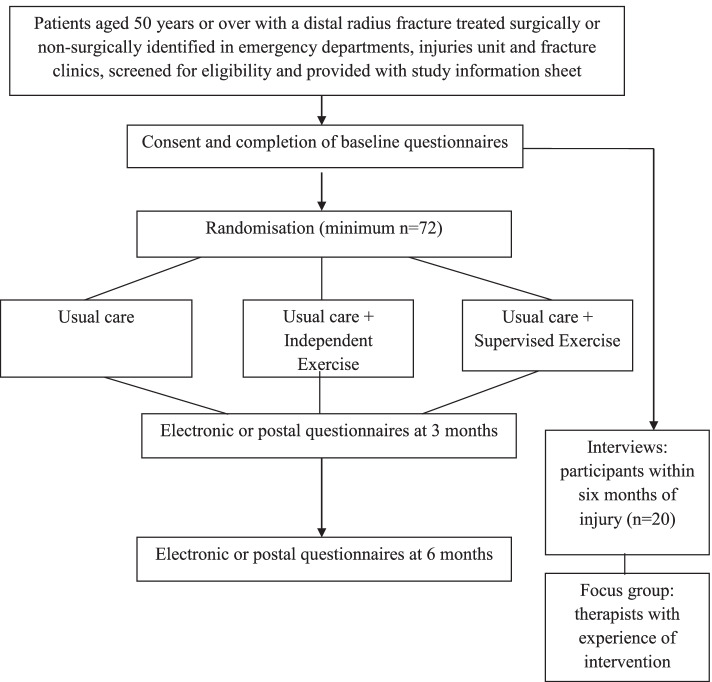
Study flow diagram

**Fig. 2:**
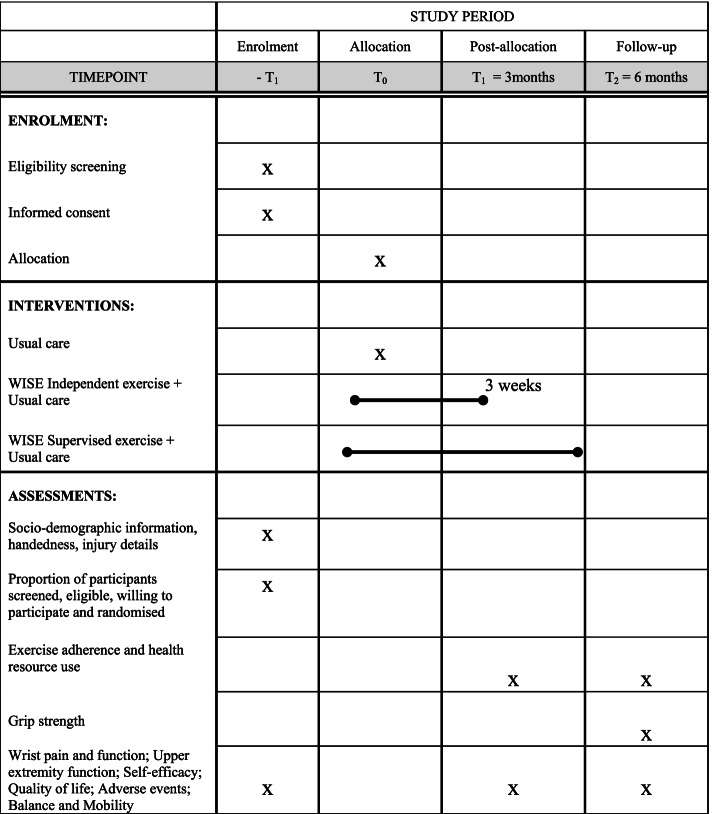
The schedule of enrolment, interventions, and assessments for the WISE trial

WISE is a multicentre, parallel, 3-group, 1:1:1, feasibility randomised controlled trial assessing two different modes of structured flexibility and resistance exercises versus usual care after distal radius fracture. The study flowchart is presented in Fig. 1. A schedule of enrolment, interventions, and assessments is presented in Fig. 2.

### Study setting

Screening, recruitment and intervention delivery will occur in at least 3 National Health Service (NHS) hospitals in the UK over a 6-month period.

### Eligibility criteria

Participants will be included if they are aged 50 years or over with a distal radius fracture treated surgically or non-surgically and willing and able to give informed consent to participate in the trial. Exclusion criteria are if the injury is more than 2 months old or there is evidence that the patient would be unable to participate in therapy appointments or the self-guided exercise programme or adhere to trial procedures (including cognitive impairment and fracture/surgery complications such as Complex Regional Pain Syndrome) or have open fractures with a Gustilo and Anderson grading > 1.

### Recruitment

Potentially eligible patient participants will be identified in the emergency department/minor injuries unit or via virtual or outpatient trauma and orthopaedic services (fracture clinic) and provided with a Patient Information Sheet. Patients that are happy to be approached about participation in the study will either be approached in the clinical setting or via telephone or video call to discuss the trial.

Screening logs recording the age, gender and initial fracture management (surgical or non-surgical), and if provided, the reasons for declining participation will be kept at each site to determine the number of patients assessed for eligibility and reasons for exclusion.

### Consent

Prior to any study-related procedures or data being collected, an online informed consent form will be completed. Permission from the participants will also be obtained to inform their general practitioner of their inclusion in the study. The person who obtains the consent will be suitably qualified and experienced and have been delegated to do so by the Chief/Principal Investigator.

After an informed consent discussion either in person or via telephone/video call, those happy to participate will provide their consent using the latest approved version of the online Informed Consent Form. If the contact is not in person and the participant does not have internet access, the consent will be recorded by a member of the local team on a Verbal Informed Consent Form during an informed consent video/telephone call and a copy sent to the participant.

### Allocation

After consent, participants will be randomised to usual care, usual care and independent exercise or usual care and supervised exercise on a 1:1:1 basis using a validated computer randomisation programme managed through a secure web-based service by the Oxford Clinical Trials Research Unit, with a minimisation algorithm to ensure balanced allocation across the three treatment groups, stratified by centre and initial fracture management (surgical vs. non-surgical). The first few participants will be randomised using simple randomisation to seed the minimisation algorithm and a probabilistic element will be introduced to the algorithm to ensure unpredictability of intervention allocation. On randomisation of a participant, the central trial office, main site contact and local study team will be notified via an automated email.

### Blinding

It will not be possible to blind the study participants or those delivering the interventions. The local research team reviewing hospital records and the trial statistician will also not be blind to the treatment allocation. The majority of outcome measures are patient-reported and collected electronically or via post, so clinicians involved in the care of patients will not be involved in outcome data collection within the trial. The research team member contacting the participants to offer support with remote hand strength measurements at 6 months after randomisation will be blinded to treatment allocation.

### Interventions

#### Usual care

On the day of cast or splint removal (usually 4 to 8 weeks after fracture), advice on self-management will be delivered by a surgeon, physiotherapist or occupational therapist in a fracture clinic, as per routine NHS clinical practice. Advice will include simple hand and wrist mobilisation exercises to restore flexibility and guidance on building up daily activity gradually. Participants may also receive a local advice leaflet, if routine practice at that hospital.

#### Independent exercise

Participants randomised to this treatment group will receive usual care and then a single physiotherapy or occupational therapy session (face-to-face or via videoconference or telephone) of up to 60 min duration no later than 3 weeks after the cast/splint removal. The purpose of this session will be to assess the participant and introduce the self-managed resistance exercise programme.

The WISE exercise programme utilises a range of progressive flexibility and resistance exercises based upon functional movements designed to promote recovery of the strength required for activities of daily living (e.g., chopping food, lifting, pushing and jar opening) and enable participants to progress their exercises after the initial set-up session with the therapist. Participants will be provided with resistance bands and putty to do the resistance exercises.

The behaviour change strategies will involve the participants identifying personal functional goals relevant to their wrist and hand function; making action plans as to where and when the home exercises will be performed; identifying barriers to exercise; and forming a contingency plan to manage difficulties in sticking to their exercises. Participants will be provided with a carefully designed, high-quality workbook that contains information on self-management, the WISE exercises, and goal planners and exercise diaries to complete. They will sign their goal planner (either on paper or electronically) during their first session with the therapist to confirm to themselves their intentions to doing exercises as there is evidence that this can facilitate engagement in an exercise programme [8].

Participants will also be given access to the WISE self-management website, if preferred over the workbook. The website content will mirror the workbook and also contain advice and exercise demonstration videos, written information and online goal planners and exercise adherence tracker via the exercise diary.

#### Supervised exercise

Participants randomised to this treatment group would receive usual care and access to the WISE exercise workbook and website and three individual therapy sessions (face-to-face or via videoconference or telephone) over 12 weeks. During the sessions, there will be opportunity for individualised feedback on progress of their rehabilitation. Therapists will focus on helping participants identify barriers to exercise and facilitate problem-solving.

#### Concurrent healthcare for all participants

Other aspects of the participant’s healthcare will be as per usual NHS local practice. In line with usual practice, participants that have difficulties with exercises will be supported over the phone/videoconference. We will carefully monitor the use of any additional contact required during the feasibility study. Participants will be able to access therapy services for post-fracture complications such as development of complex regional pain syndrome according to usual local routes of referral. The use of out of trial therapy will be captured in follow-up questionnaires.

### Primary feasibility outcomes

#### Patient engagement with the trial

The number of eligible participants who are screened, eligible and randomised will be collected to estimate the recruitment rate and proportion of patients willing to take part in the future trial.

#### Acceptability of the interventions

We will assess the acceptability of the interventions by therapist and participants’ adherence and their experiences with the WISE interventions.

Treating therapists’ adherence to the intervention protocol will be assessed by (1) number of therapy sessions provided by the therapists, (2) content of intervention sessions and (3) timing of sessions. For complete adherence, we expect one session to be provided and attended for the independent exercise group and three for the supervised exercise group. During each session, all core intervention components will need to be delivered. The first session will need to be delivered as soon as possible, at least within 3 weeks of cast/splint removal. Additionally, for supervised resistance exercise, all three sessions must be completed within 12 weeks from the first to last session.

In terms of participant adherence to exercise, we will record the proportion of participants who report performance of resistance exercises at home and use the exercise support materials (website and workbook).

As part of the feasibility evaluation, we will also conduct qualitative interviews with participants and a focus group with the treating therapists to understand their experience of the trial and trial interventions. Participant interviews will be undertaken via telephone/video call or face-to-face in order to understand the experience of (i) the intervention within the context of their daily life, (ii) what taking part in a randomised trial is like, and (iii) outcomes that are important to them. Up to 20 participants will be interviewed within 6 months of injury. Participants will be purposively sampled to ensure representation from each intervention group, including surgical and non-surgical management of their wrist. Patients who decline to take part in the randomised study will also be interviewed to explore the experience of (i) recovery from injury, thoughts and feelings about the intervention, what helped or hindered their recovery; (ii) thoughts and feelings about taking part in the trial and trial processes; and (iii) what they hope to achieve and challenges they have or may encounter.

Up to 15 therapists involved in delivering the trial interventions will be invited to participate in a face-to-face or online focus group to be held towards the end of the treatment phase to explore their experience of (i) delivery and fidelity of the interventions, (ii) what helped or hindered the patient’s ability to undertake the intervention and (iii) contextual factors that helped or hindered the trial. Interviews and the focus group will be digitally audio-recorded and transcribed verbatim. Analysis will be through coding sections of data, drawing codes with similar meanings together to form categories and themes. NVivo, a qualitative software package [9], will be used to manage the data.

#### Retention of participants in the trial

We will review the proportion of participants who complete and return their 3 and 6 month follow-up questionnaires. In addition, we will record the number of patients who complete and return grip strength measurements at 6 months.

### Progression criteria 

The feasibility objectives from our quantitative evaluation and their respective progression criteria are summarised in Table 1. Progression criteria to assess feasibility of a future definitive trial will be assessed using a traffic light system [10]. ‘Green’ indicates feasible with current procedures, ‘Amber’ indicates modification to one or more components of the protocol is required in order to proceed and ‘Red’ indicates a definitive trial would not be considered feasible.

**Table 1:** Feasibility objectives and progression criteria

	Green (feasible)	Amber (modify)	Red (not feasible)
Feasibility objective 1: Patient engagement with the trial			
Recruitment acceptability	≥ 50% of eligible patients screened are consented and randomised	20–50%	< 20%
Recruitment rate	≥ 4 participants recruited per recruiting month per centre	1–3 per month per centre	< 1 per month per centre
Feasibility objective 2: Acceptability of the interventions			
Intervention adherence	> 75% of participants receive the allocated intervention sessions as per protocol	55–75%	< 55%
Feasibility objective 3: Retention of participants in the trial			
Proportion of randomised patients providing outcome data at 6 months follow-up	< 20% loss to follow-up at 6 months (including deaths and withdrawals)	20–30%	> 30%

### Secondary exploratory outcomes

We will also collect the following exploratory outcomes as part of this feasibility study to determine their viability for inclusion in a future definitive trial.

- Wrist pain and function: Wrist pain and function measurement using the Patient Reported Wrist Evaluation (PRWE) [11] recommended by the Core Outcome Measures in Effectiveness Trials (COMET) guidance [12] for distal radius fractures will be followed. PRWE is a 15 item patient-reported questionnaire that assesses pain and functional difficulties in activities of daily living resulting from injuries affecting wrist joint area. The pain subscale has 5 items (each rated 0 = no pain to 10 = worst pain) and the function subscale has 10 items (each rated 0 = no difficulty to 10 = unable to do). Total score ranges from 0 to 100, higher scores indicate worse wrist pain and function.
- Upper extremity physical function: Patient Reported Outcome Measurement Information System (PROMIS) [13] Physical Function (Upper Extremity) questionnaires will be administered electronically. They are a Computer Adaptive Test, which are dynamic tests based on Item Response Theory. A mathematical model adapts the sequential questions asked based on a participants’ previous response. A tailored set of questions is therefore asked from a large item pool. PROMIS instruments are scored from 0 to 100 with 50 points representing the mean score for the US general population, higher scores indicate better function. This questionnaire has been found to be valid in the context of upper limb fractures in the UK [14, 15]. Participants with no Internet access will be able to complete a paper-based version of the questionnaire (PROMIS Upper Extremity Short Form, 7a).
- Self-efficacy: The Self-Efficacy for Exercise Scale [16] is a 9-item questionnaire (total scores range from 0 to 90, higher scores indicate higher self-efficacy for exercise) that will be used to assess participants’ confidence in their ability to do exercise.
- Exercise adherence: Participants will be asked to indicate how many times in the preceding week they have done specific exercises for their injured hand and upper extremity, to assess engagement with the advised exercises after wrist fracture.
- Quality of life: The EuroQol 5 Dimensions (EQ-5D-5L) is a validated, generalised and standardised instrument comprising a visual analogue scale (VAS) measuring self-rated health and a health status instrument, consisting of a five-level response (no problems, some problems, moderate problems, severe problems and unable) for five domains related to daily activities: (i) mobility, (ii) self-care, (iii) usual activities, (iv) pain and discomfort and (v) anxiety and depression [17]. Responses to the health status classification system are converted into an overall score using a published utility algorithm for the UK population. The EQ-5D health status scale ranges from negative scores − 0.594 [reflective of a patient’s quality of life being worse than death], 0 [death], to 1 [perfect health]. A respondent’s EQ-VAS gives self-rated health on a scale where the endpoints are labelled ‘best imaginable health state’ (100) and ‘worst imaginable health state’ (0).
- Health resource use: A bespoke health resource use questionnaire will be used to assess the number of primary and secondary care consultations, additional therapy appointments, further wrist x-rays and scans, surgery and over-the-counter pain medication prescribed, out-of-pocket expenses and work absence.
- Adverse events: Adverse events related to the randomised interventions will be recorded.

A serious AE (SAE) is any unexpected untoward medical occurrence relating to the trial interventions resulting in death, is life-threatening, requires inpatient hospitalisation or prolongation of existing hospitalisation or persistent or significant disability/incapacity. In the event of any SAE, the Clinical Trials Unit standard operating procedures will apply.

Foreseeable SAEs and adverse events not defined as serious that are related to the interventions will be recorded by participants or site staff but will not need to be reported immediately. These events will be recorded on patient-reported questionnaires or by the site investigators in the ‘Complications’ case report form if they become aware of such an event.

Foreseeable adverse events include:

- Increases in pain lasting more than 1 week
- Treatment-related exacerbations of other medical conditions that do not meet the definition of serious (for example angina after exertion)
- Development of Complex Regional Pain Syndrome
- Surgery to the injured wrist (unless an adverse event directly related to the exercise intervention, in which case this would be an SAE)

Safety reporting will begin from the point of randomisation and end when the participant has reached their final main follow-up time point, at 6 months post-randomisation.

- Muscle strength of the upper limb: Force produced during cylindrical grip will be measured using a commercially available hand-held dynamometer in a sitting position, as recommended by the American Society for Hand Therapists [18, 19]. Three attempts will be recorded for each hand alternatively, starting from the uninjured side on two consecutive days. Participants are asked to wait a minimum of 30 s between recording measurements. The best grip score for each hand will be recorded.
- Balance and mobility: Participants will be asked Likert type questions on balance and mobility to assess how this is progressing since their injury.

### Sample size determination

In a definitive trial with two intervention groups and with the PRWE as primary outcome, a sample size of 486 participants would be needed, based on a standardised effect size of 0.33, power of 90%, an alpha error of 0.05 (2-sided) and inflating for 20% loss to follow-up. The effect size equates to a clinically meaningful difference of 6 points in the PRWE, and a standard deviation of 18 which is consistent with evidence from other cohorts [20–22]. Currently, we do not know whether it is feasible to conduct a definitive trial. To recruit 486 participants over 20 months in the definitive trial at six recruitment centres, we would need to recruit at least four participants per centre per month. In order to improve the precision of the estimate of the number of participants recruited per month we will recruit over a six month period. If we recruit on average four participants per centre per month we will recruit 72 participants at three feasibility study centres in a six month period.

### Data collection methods

#### Baseline data

Baseline sociodemographic, handedness, injury details will be collected. Participants will also complete the PRWE, PROMIS Upper Extremity, EQ-5D-5L, balance and mobility and Self-efficacy Exercise Scale.

#### Treatment logs

After the usual care or exercise (independent and supervised) sessions, the date, duration, session content, clinician profession and experience details, setting, mode of delivery and the material and resources issued will be recorded on treatment logs.

#### Three- and six-month follow-up

Participants will receive an electronic/paper invite to complete questionnaires which include the PRWE, PROMIS, EQ-5D-5L, self-efficacy to exercise, current balance and mobility status, exercise frequency, resource use and adverse events. Reminders will be sent by email, post and/or text message (according to the participant’s preference); if questionnaires are not completed, these will be followed by a phone call from the central team if required.

#### Six-month follow-up grip strength measurement

Only those participants who return the 6-month follow-up questionnaires will then immediately be sent a portable dynamometer and a grip recording form with written instructions on how to use the device and given access to a video guide. A member of the research team via telephone or video call will contact participants to offer the support to do the measurements if required. Participants will be provided with free-post return packaging to return the grip data and dynamometer.

#### Early discontinuation/withdrawal of participants

During the course of the trial, a participant may choose to withdraw early from the study at any time, without giving reasons, and without prejudicing their clinical care. Participants will not have the option to withdraw the data collected up until the point of withdrawal, as the data will be required for the intention-to-treat (ITT) main analysis and analysis of safety. The type of withdrawal and reason for withdrawal, if the participant is willing to provide one, will be recorded. In addition, therapists may discontinue a participant from the study treatment at any time if necessary to safeguard the safety or wellbeing of the participant, including but not limited to ineligibility (either arising during the study or retrospectively having been overlooked at screening). Withdrawn participants will not be replaced.

#### Data management

At enrolment, participants will be asked to indicate their preference for the delivery and completion of questionnaires—electronic or postal follow-up at 3 and 6 months. Data collected in electronic format will be entered directly onto the trial database, including the collection of documentary evidence of consent. All data entered will be encrypted in transit between the participants/recruitment centre and server. All electronic patient-identifiable information will be held on a server located in an access-controlled server room at the University of Oxford. The data will be entered into a Good Clinical Practice (GCP) compliant data collection system and stored in a database on the secure server, accessible only to the research team based on their role within the study. The database and server are backed-up to a secure location on a regular basis.

Identifiable data will be limited to contact details and will be accessed separately from the outcome data obtained from/about the participants and managed within the rules of the clinical database system. Direct access to source data/documents will be required for trial-related monitoring and/or audit by the Sponsor, NHS Trust or regulatory authorities. Contact details will be retained for 12 months after the last data collection. Electronic de-identified trial data will be retained for 3 years after publication of the trial findings. Site staff will have access to the centrally collected patient-reported outcome data for participants that they recruit at their site on Research Electronic Data Capture (REDCap), to ensure that they can download a complete dataset for their patients at the end of the trial.

### Statistical methods

Analysis methods will be guided by the demonstration of feasibility. If feasibility is not demonstrated and a definitive trial is not conducted, then outcome data for the ITT population will be analysed and reported for each randomised group. In this case the feasibility outcomes and baseline characteristics will be reported using descriptive statistics. Mean and standard deviation or median and interquartile range will be used for continuous variables and counts and percentages will be used for any binary or categorical variables. Missing data will be minimised by careful data management. No comparative statistical testing will be undertaken as this is a feasibility study and is not powered for this purpose.

If feasibility is demonstrated, outcome data will not be analysed by group as we would take participants and their data from the feasibility and pilot study forward into the definitive trial.

### Data monitoring

Quality control procedures will be undertaken during the recruitment and data collection phases of the study to ensure research is conducted, generated, recorded and reported in compliance with the protocol, GCP and ethics committee recommendations. The Chief Investigator and the Trial manager will develop data management and monitoring plans. The day-to-day management of the trial will be the responsibility of the Trial Manager, supported by a Senior Trial Manager. This will be overseen by the Trial Management Group (TMG), who will meet monthly to assess progress. The Trial Manager will also ensure training of the research staff at each of the trial centres is undertaken. The trial statistician and the information specialist will be closely involved in setting up data capture systems, design of databases and clinical reporting forms. The TMG will maintain robust oversight of trial conduct and safety.

This study will be coordinated by the UK Clinical Research Collaborative registered Oxford Clinical Trials Research Unit (OCTRU) at the University of Oxford. A rigorous programme of quality control will be implemented to ensure compliance to the current approved protocol, GCP, relevant regulations and OCTRU Standard Operating Procedures (SOPs). Quality assurance checks will be undertaken by the trial management team to ensure integrity of randomisation, study entry procedures and data collection. Inspections of the Trial Master File will be carried out by the OCTRU Quality Assurance team (at least once in the lifetime of the study, more if deemed necessary). Furthermore, the processes of consent taking, randomisation and registration, provision of information and provision of treatment will be monitored centrally.

Intervention delivery will be from study trained therapists and will be monitored periodically to ensure fidelity. Site visits and/or audio/video recording of interventions will be conducted. Permission will be sought from the trial participants to observe or record treatment sessions. Verbal consent will be provided and recorded. Case Report Forms will also be used to monitor intervention fidelity. Data will be collected on intervention content delivery and number of treatment sessions attended to facilitate monitoring and reporting. The sites will regularly receive feedback from quality activities to help maintain and improve fidelity. Additionally, the study may be monitored, or audited by sponsor or host sites in accordance with the current approved protocol, GCP, relevant regulations and standard operating procedures.

### Ethics and dissemination

This study was approved by the South Central - Hampshire B Research Ethics Committee, ref: 20/SC/0433. This protocol has been reported following the Standard Protocol Items: Recommendations for Interventional Trials (SPIRIT) statement [23].

Results will be published in a peer-reviewed journal with authorship eligibility according to the International Committee of Medical Journal Editors criteria. The final report will detail amendments to the study protocol. We will work with networks to disseminate findings, for example, through annual meetings and newsletters of the Association of Trauma and Orthopaedic Chartered Physiotherapists, Orthopaedic Trauma Society and the Global Fragility Fracture Network. A plain language summary of the results will be emailed to the trial participants. The findings will be shared with patients and the public more widely through local and national charity newsletters and other media channels. Social media will be utilised to share news on study progress. We will be supported in our dissemination by the Oxford NIHR Biomedical Research Centre communications officer.

### Patient and public involvement

Two patient representatives (RG and JG) are co-investigators of this trial and are integral members of the regular TMG meetings. The UK Musculoskeletal Trauma Patient and Public Involvement (PPI) group were directly involved in the development of the research question and planning of the trial. They provided feedback and comments in producing the patient facing materials including the informed consent form, study information sheet and self-management support materials (workbook and website). The PPI members of the TMG will also be involved in a co-analysis day to discuss the themes evolving from the interviews and focus group to draw out the aspects of the WISE interventions that either help or hinder recovery in people with distal radius fracture.

## Discussion

This feasibility trial assesses a structured flexibility and resistance exercise programme (WISE) delivered with behaviour change strategies, targeted to improve functional recovery through performance of regular progressive exercise. The exercise programme will be delivered through independent and supervised modes. This model allows self-guided management and regular exercise at home by patients.

The COVID-19 pandemic resulted in important changes in the planned study design prior to finalising the study protocol. A planned face-to-face follow-up clinical assessment visit at the recruiting hospital at 6 months to undergo a range of strength measurements was altered. Instead, a questionnaire was sent, with those who respond being sent a hand-dynamometer to test and record their grip strength. We also added the option for remote consent and intervention delivery to enable the flexibility in delivery required in the context of reduced face-to-face healthcare contact. The changing clinical environment will be an important factor to consider when analysing and interpreting the feasibility data.

This feasibility trial has several strengths. This is the first trial to evaluate the feasibility of a structured exercise programme using both quantitative data collection methods and an embedded qualitative study. This trial will add to the existing literature on the overall adherence, feasibility and participant and therapists’ experiences of receiving and delivering the WISE programme in both modes. The WISE programme was developed from the core components of the evidence-based Strengthening And Stretching for Rheumatoid Arthritis of the Hand (SARAH) programme, recommended by the National Institute for Health and Care Excellence guidelines for people with rheumatoid arthritis of the hand [24, 25]. We also have significant input from patient and public representatives, and therapists who treat upper limb conditions (see Acknowledgements). We have designed the WISE exercise programmes, supplemented with behaviour change strategies such as goal planning and self-monitoring, to enhance therapeutic impact and improve clinical outcomes in this patient population. Therapists who delivered the exercise programme will be trained through standardised remote live training sessions or online training modules on the content and delivery of the programme and reporting of treatment sessions.

There are some limitations, the main one being that with a limited number of sites it will be challenging to assess feasibility across the NHS; however, we are purposively working with recruitment sites that represent a range of geographical and clinical settings (major trauma centres and trauma units).

If this feasibility trial is successful, it will guide the development of a definitive trial across the UK. In turn, this could lead to recommendations about future routine NHS care practice for patients with this injury. Trial recruitment is on-going at the time of manuscript submission.

## Data Availability

Not applicable.

## Abbreviations

AE: Adverse event
EQ-5D-5L: EuroQol 5 Dimensions 5 Levels
GCP: Good clinical practice
ITT: Intention to treat
NHS: National Health Service
OCTRU: Oxford Clinical Trials Research Unit
OR: Odds ratio
PPI: Patient and public involvement
PROMIS: Patient Reported Outcome Measurement Information System
PRWE: Patient Reported Wrist Evaluation
REDCap: Research Electronic Data Capture
SAE: Serious adverse event
SARAH: Strengthening And Stretching for Rheumatoid Arthritis of the Hand
SOP: Standard operating procedure
SPIRIT: Standard Protocol Items: Recommendations for Interventional Trials
TMG: Trial management group
WISE: Wrist injury strengthening exercise
